# Force Transmission Analysis and Optimization of Bowden Cable on Body in a Flexible Exoskeleton

**DOI:** 10.1155/2022/5552166

**Published:** 2022-07-28

**Authors:** Xin Li, Jinkang Liu, Weihao Li, Yijing Huang, Gan Zhan

**Affiliations:** ^1^School of Mechanical and Materials Engineering, North China University of Technology, Beijing 100144, China; ^2^School of Mechatronics Engineering, Beijing Institute of Technology, Beijing 100081, China

## Abstract

The Bowden cable is a significant force transmission equipment for a flexible exoskeleton. However, the previous researches of Bowden cable had emphasized on the data from experimenting test board, instead of on human body, which produced the inaccurate assisting analysis of the flexible exoskeleton. In this paper, a flexible exoskeleton for assisting knee extension was proposed, which provided an on-body condition. Then, the friction force and its influencing factors between the wire rope and sheath of the Bowden cable from the motor to the anchor of knee have been analyzed. The segment models of force transmission with the concern of three kinds of friction modes were established, and the relationship between various lengths and bending angles of Bowden cable was fitted to the equations of curve. Furthermore, the association rule between the force transmission and the lengths of Bowden cable was obtained, based on which, the optimal force transmission efficiency was 78.68% when the length value of the Bowden cable was 475 mm. A flexible exoskeleton prototype was assembled; then, the experiments with force transmission and metabolic cost have been developed. The results showed that the force transmission efficiency had strong association with the lengths of Bowden cable, instead of the transmission velocities. Furthermore, this knee assistance exoskeleton reduced the net metabolic cost of the testees during walking. These experiments results corroborated the force transmission modeling and simulation of the Bowden cable on body we proposed in this paper.

## 1. Introduction

Exoskeleton is a kind of intelligent wearable robot that can assist the upper or lower limbs of human movement. With the development of its technology, exoskeletons have been widely used in medical rehabilitation, logistics, and military fields [1–5]. Based on the structure mode, exoskeletons are divided into rigid assist [6–8] and flexible assist [9–14]. The rigid exoskeleton is composed of several rigid connecting rods, which are bound to the human limbs tightly. Although this kind of exoskeleton can transfer the weight of load to the ground, it also creates the resistance to the normal movement due to the joints' axis inconsistency of the exoskeleton and human limbs.

In recent years, many researchers have moved the focus to flexible exoskeletons due to the defects and boundness of rigid exoskeleton. The type of flexible exoskeleton can be divided into three modes: pneumatic muscle assist, smart material assist, and Bowden cable assist [15]. The mode of pneumatic muscle-based assistance requires high air pressure, which brings air leakage risks, making it dangerous for human wear. The research and application of the smart materials for exoskeleton are still in the basic stage, and this technology is under developing. In contrast, Bowden cable has been widely used to drive flexible exoskeletons due to its advantages of light weight, small size, and strong flexibility. The below references illustrated the Bowden cable application for flexible exoskeleton. Awad et al. [16] have developed a soft robotic exosuit that improved walking in patients after stroke. It has been proved that this system can contribute to a 20 ± 4% reduction in forward propulsion interlimb asymmetry and a 10 ± 3% reduction in the energy cost of walking. Ding et al. [17] have proposed an IMU-based iterative control method for hip extension assistance with a soft exosuit, and the metabolic reductions ranging from 5.7% to 8.5% were found when comparing the powered conditions with the unpowered condition. In the follow-up research, their team optimized the controller of the system and introduced the Bayesian algorithm to identify the peak and offset timing of hip extension assistance that minimizes the energy expenditure of walking with a textile-based wearable device. The system was also proven to reduce the wearer's metabolic value by 17.4% ± 3.2% compared with walking without the device [18, 19]. Jin et al. [20] designed a cable-driven active leg exoskeleton, which employed the “assist-as-needed” control strategy to help the ankle center move along a prescribed path. Nine healthy subjects were trained by a cable-driven leg exoskeleton to walk in a new gait pattern, and the gait of the subjects became significantly closer to the target gait after 40 minutes of training [21]. Mooney et al. [22] developed a flexible assisted exoskeleton for the ankle joint under load carriage, in which the wearer carried a weight of 23 kg and walked on a treadmill at a speed of 1.5 m/s. Compared with the condition without wearing the exoskeleton, the metabolic cost of walking reduced by 36 ± 12 W. Gasparri et al. [23] have developed an ankle-assisted flexible exoskeleton system for patients with pathologic gait due to cerebral palsy. Patients wearing the device can reduce the net metabolic rate of the ankle joint by 16% during walking. In the follow-up study, the control strategy by proposing a proportional controller based on joint torques was improved, which helped the wearer reduce metabolic values by 17% to 27% compared to the unassisted condition [24]. Schmidt et al. [25] introduced a soft wearable exosuit-“Myosuit,” which can provide continuous assistance at the hip and knee joints when working with and against gravity in ADLs. In addition, the system can provide successfully identify changes in the posture and assist hip and knee extensions with up to 26% of the natural knee moment and up to 35% of the knee power. Natali et al. [26] have developed a soft exoskeleton-“XoSoft,” a modular exoskeleton system for lower limbs, to help people with mobility problems. The system has been proven to help the wearer reduce energy consumption between 10% and 20%. Cao et al. promoted various kinds of soft exoskeletons for hip assistance, such as the rigid-soft structure combination [27], the proportional derivative iterative learning controller [28], and a novel hardware circuits design [29]; furthermore, the results of the net metabolic cost with three kinds of methods above presented a certain amount of decline. Wu et al. [30] developed a knee energy harvesting exoskeleton that can generate electricity during level walking, downhill walking, and stair descent without sensors, which generate enough power while has little metabolic effect on the wearer. Meanwhile, different control strategies based on gait segmentation and driving elements were also studied, which allowed the exoskeleton to improve gait and posture during specific phases of the human walking cycle. According to the references above, we can conclude that the Bowden cable-based drive has been widely used in flexible exoskeletons and provided the effective assistance and less metabolic cost. However, the key weakness of the Bowden cable drive exists in the friction between the internal wire rope and the external sheath, which will reduce the performance of the transmission system. Although many researchers have put emphasis on the assistance modes of Bowden cable for the joints of limbs and metabolic rate of the tests, there are less attention on how to improve the efficiency by optimizing the layout of Bowden cable. Therefore, it is necessary to establish the model of Bowden cable, then optimize the force transmission system of the flexible exoskeleton by analyzing the friction elements, so as to improve the force transmission efficiency and enable the flexible exoskeleton functioning optimally.

According to the analysis of the Bowden cable, several scholars have carried out some research on the modeling of Bowden cable transmission system to analyze its characteristic. Chen and Wang [31] derived the torque and displacement transfer model of the coupling transmission system of the double Bowden cable and established a test platform for verification. The result showed the transmission characteristics are determined by the total sheath bending angle, tendon pretension, and the friction level between sheath and tendon. Agrawal et al. [32] analyzed the loss of force transmission due to the driving wire and the sheath of the surgical robot. A model of double Bowden cable force transfer in continuous time domain was established by partial differential equation and verified by experiments. Jeong and Cho [33] proposed a novel method of compensating for the changing nonlinearity of the Bowden cable, which enabled controlling the tension of a Bowden cable without directly measuring the output tension. The results showed that the control error of the output tension decreases from 5.21 to 0.50 N when the bend angle of the Bowden cable changes from 0° to 400°. Wang et al. [34] presented a series of new developments to improve the performance of the robot driven by tendon-sheath system: a force transmission model to address flexibility, elongation study for precise position control, and tissue property modeling for haptic feedback. Furthermore, they also proposed a system modeling approach for motion compensation, which is evaluated by trajectory tracking under different environment loading conditions [35]. These studies above showed the effective force transmission of the Bowden cable and analyzed the friction between the wire rope and sheath. However, the main contributions were based only on the results from experiment platforms, without consideration of the impact from structure characteristic of the exoskeleton and the movement of the human body. It can be concluded that a novel friction model combined with the limbs' movement of the Bowden cable should be established.

To address the above needs, the force transmission of the Bowden cable in a flexible exoskeleton on body was analyzed and optimized in our paper. The main contributions of this paper are as follows: (1) demonstrating that the Bowden cable form on body is a key factor to influence the force transmission of the exoskeleton; (2) analyzing three kinds of friction mode in Bowden cable, and optimizing the length of Bowden cable to obtain the maximum force transmission efficiency; (3) conducting the force transmission and metabolic cost experiments to evaluate the effectiveness of this flexible exoskeleton and its Bowden cable optimization.

The research contents are as follows: first, a mode of lower limb exoskeleton with Bowden cable was designed, based on which, the force transmission models of Bowden cable were established with the three kinds of friction factors under the whole transmission process. Second, the variation rules and the maximum force transfer efficiency were obtained by the calculation and simulation with various lengths of the Bowden cable. Finally, the force transmission and metabolic cost experiments were conducted to verify the force transmission models and efficiency calculation, which could provide a more efficient power assistance and less metabolic cost.

## 2. Materials and Methods

**Figure 1:**
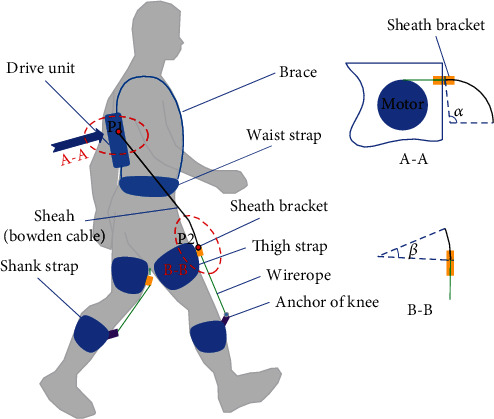
Sketch of a flexible exoskeleton for the knee extension.

According to the difference between the flexible exoskeleton and rigid exoskeleton design, Bowden cable transmission has taken place of the rigid rod, on the other hand, the energy driving system which needs to be designed as concentrated and miniaturized. Our flexible exoskeleton for assisting knee extension is designed as Figure 1. It is mainly composed of drive unit, Bowden cable (including sheath and wire rope), thigh strap, shank strap, brace, waist strap, sheath bracket, and anchor of knee. The system works as follows:

1. The function of brace and waist strap is to fix the drive unit on the human back
2. The thigh strap and shank strap are to bind with trousers tightly for reducing the deformation which may bring the loss of force and assistance delay
3. The function of the sheath bracket is to restrain the sheath and allow the wire rope pass through the bracket; furthermore, it can constrain the direction of the Bowden cable, which transfers force along the sagittal plane of the thigh
4. The drive unit provides force for wire rope moving relative to the sheath, and the force acts on the anchor of the knee for its stretching movement

When the flexible exoskeleton provides assistance for the extension of knees, the Bowden cables stretch out from the two sides of the drive unit and pass through the sheath brackets, then exert force on the anchors of knees. In this process, the angle *α* and *β* appear on the Bowden cable. The angle *α* exists on the Bowden cable from the sheath bracket in the drive unit, and the angle *β* exists on the Bowden cable entering the sheath bracket in front of the thigh. The different *α* and *β* may bring various friction between the sheath and wire rope of the Bowden cable. Furthermore, the different lengths of the Bowden cable from P1 to P2 can produce various combinations of *α* and *β*. In this paper, our aim is to optimize the length of the Bowden cable and obtain the optimal force transmission efficiency.

### 2.1. Force Transmission Model of Bowden Cable on Body

In the process of force transmission with Bowden cable, the main friction is created between the sheath and wire rope; furthermore, the frictional loss will influence force transmission efficiency. Force transmission analysis of Bowden cable has been always considered in static condition with experiment platform [31–33, 36]; however, there are some differences in wearable condition of Bowden cable on body: first, the extrusion between the sheath bracket and Bowden cable will produce a different friction, what is more, the friction between the wire rope and the sheath bracket working with the swing of thigh produces different effects when the human body moves. Therefore, in order to obtain the accurate efficiency of Bowden cable on body, a novel force transmission model of Bowden cable on body should be established and analyzed.

**Figure 2:**
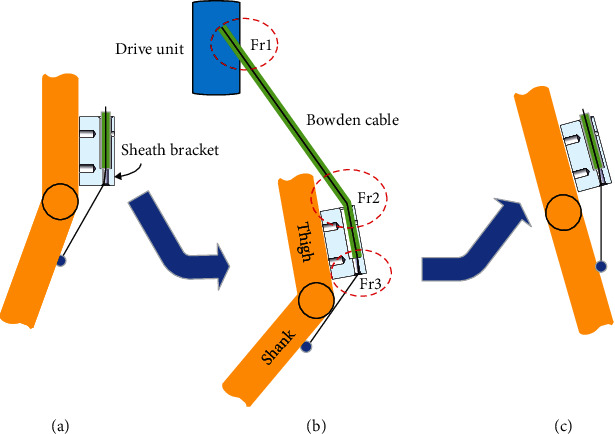
The swinging phase and friction locations of Bowden cable. (a) The beginning position of the swing phase. (b) The ultimate state of the swing phase. (c) The end position of the swing phase.

According to above analysis, the various friction not only exists between the wire rope and its sheath of the Bowden cable in free state or in extrusion with sheath brackets but also exists between the wire rope out of the sheath and the sheath bracket. Furthermore, the friction between the wire rope and the sheath bracket changes along with the different phases in the process of walking. In this paper, the maximum friction in the walking gait will be considered in the calculation of the efficiency of the Bowden cable force transmission. The variation of knee's angles occurs in swinging phase of the gait, and Figure 2 showed the whole swinging phase. As we can see, the maximum friction appeared when the knee joint is at the ultimate state of the swinging phase (see Figure 2(b)). The force transmission model should be established in this state, and the main friction places Fr1, Fr2, and Fr3 were indicated in Figure 2(b).

**Figure 3:**
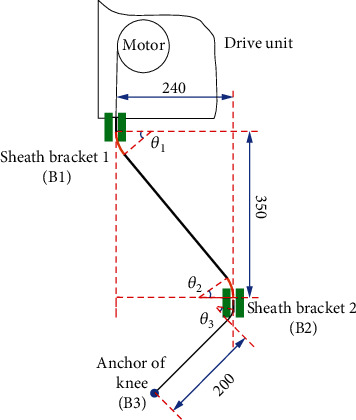
The sketch of 2-D form of Bowden cable on body.

From Figure 1, we can see that the form of Bowden cable between the drive unit and the sheath bracket in front of the thigh presents a spatial variation, which has two curve angles. To simplify the calculation in 3-D space, the curve of Bowden cable was converted to 2-D form, which considered the length variation rule and its characteristics of the Bowden cable on the wearer.  Figure 3 presented a 2-D form of the Bowden cable, which originated from the sheath bracket 1 of the drive unit to the sheath bracket 2 in front of the thigh, and the wire rope was driven by motor, then forced on the anchor of knee through the sheath. The width value from the sheath bracket1 (B1) to the sheath bracket2 (B2) is 240 mm, and the height value from B1 to B2 is 350 mm under the measurement and simulation. The length of the wire rope from B2 to the anchor of knee (B3) is 200 mm, which is under the ultimate state of the swinging phase. Furthermore, there are three angles *θ*_1_, *θ*_2_, and  *θ*_3_ existing in the curve of Bowden cable.

**Figure 4:**
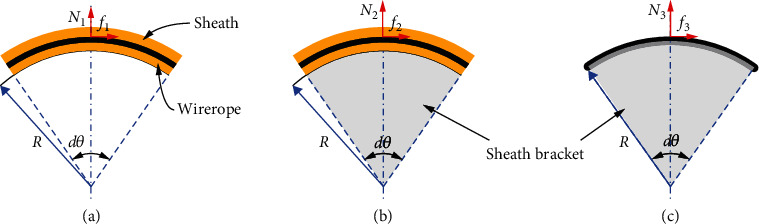
The three sketches of the friction and pressure of Bowden cable. (a) The friction of Bowden cable in free state. (b) The friction of Bowden cable in extrusion state. (c) The friction between the wire rope and sheath bracket 2.

As Figure 3 suggests, the friction between the wire rope and sheath varies depending on the lengths of Bowden cable from B1 to B2. The extrusion between the Bowden cable and sheath bracket produces the most friction to the force transmission when the Bowden line is short enough. Along with the length of Bowden cable increases, the extrusion gradually decreases, and the friction between wire rope and sheath in free state of Bowden cable arises, which has a different friction factor. On the other hand, the friction between wire rope and sheath bracket 2 always exists in this process. Based on the analysis above, there exist three kinds of friction modes (see Figure 4): (a) the friction *f*_1_ and pressure *N*_1_ are between wirerope and sheath in a free state of Bowden cable; (b) the friction *f*_2_ and pressure *N*_2_ are between wirerope and sheath under the extrusion of the sheath bracket to the Bowden cable; (c) the friction *f*_3_ and pressure *N*_3_ are between wirerope and sheath bracket.

**Figure 5:**
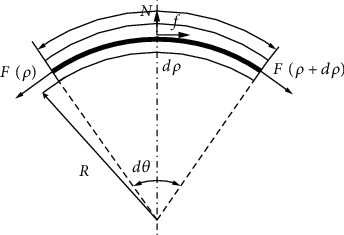
The infinitesimal model of the force transmission.

As we can see from Figure 4, the curve of Bowden cable and the pressure of extrusion are the main elements for friction. However, the three friction modes in force transmission are similar, so the basic infinitesimal model of the force transmission should be established as follows (see Figure 5).

(1) $$\begin{array}{c} \left\{\begin{array}{c} f+F\left(\rho +d\rho \right)\cos\left(\frac{d\theta }{2}\right)-F\left(\rho \right)\cos\left(\frac{d\theta }{2}\right)=0,\\ F\left(\rho \right)\sin\left(\frac{d\theta }{2}\right)+F\left(\rho +d\rho \right)\sin\left(\frac{d\theta }{2}\right)-N=0, \end{array}\right. \end{array}$$

where *N* is the pressure between sheath and wirerope, *f*  is the friction force between sheath and wirerope, *F*(*ρ*) is the tensile force of left side of infinitesimal wire rope, *F*(*ρ* + *dρ*) is the tensile force of the right side of infinitesimal wire rope, *dθ* is the angle of the infinitesimal Bowden cable, *R* is the radius of the infinitesimal Bowden cable, and *dρ* is the arc length of the infinitesimal Bowden cable.

Equation (1) can be transformed to Equation (2) as follows:

(2) $$\begin{array}{c} \left\{\begin{array}{c} f=-dF\left(\rho \right)\cos\left(\frac{d\theta }{2}\right),\\ N=2F\left(\rho \right)\sin\left(\frac{d\theta }{2}\right)+dF\left(\rho \right)\sin\left(\frac{d\theta }{2}\right). \end{array}\right. \end{array}$$

When the infinitesimal model of Bowden cable is small enough, we can consider *dF*(*ρ*)sin(*dθ*/2) as infinitesimal of higher order, then sin(*dθ*/2) ≈ *dθ*/2, cos(*dθ*/2) ≈ 1. Suppose Δ*F*(*ρ*) is the difference value between the right side and left side of the infinitesimal Bowden cable, then

(3) $$\begin{array}{c} \left\{\begin{array}{c} f=-\Delta F\left(\rho \right),\\ N=F\left(\rho \right)d\theta . \end{array}\right. \end{array}$$

According to the coulomb friction model, the friction force is as follows:

(4) $$\begin{array}{c} f=\mu N\mathrm{sign}\left(\lambda \right), \end{array}$$

where *μ* is the friction factor between the wirerope and sheath, and *λ* is the velocity vector of the wirerope relative to the sheath. Then, Equation (4) was substituted into Equation (3), and the Equation (3) can be changed as follows:

(5) $$\begin{array}{c} \frac{\Delta F\left(\rho \right)}{F\left(\rho \right)}=-\mu \mathrm{sign}\left(\lambda \right)d\theta . \end{array}$$

Then, we suppose *F*′(*ρ*) as the derivative of tension difference Δ*F*(*ρ*)  with respect to the *dρ*, and *dθ* = *dρ*/*R* = *kdρ*, where *k* is the curvature of the infinitesimal Bowden cable. Equation (5) can be changed as follows:

(6) $$\begin{array}{c} \frac{F^{\prime }\left(\rho \right)}{F\left(\rho \right)}=-\mu \mathrm{sign}\left(\lambda \right)kd\rho . \end{array}$$

We suppose the curvature *k* as a constant value, then *θ* = ∫_0_^*l*^*kdρ*, and set *F*_in_ and *F*_out_ as the input force and output force of the infinitesimal Bowden cable. Equation (6) can be solved by integration as follows:

(7) $$\begin{array}{c} F_{\text{out}}=F_{\text{in}}e^{-\mu \theta \mathrm{sign}\left(\lambda \right)}, \end{array}$$

where *l* is the length of the Bowden cable, and *θ* is the curve angle of the Bowden cable.

Equation (7) shows that the transmission force loss depends on the friction factor and curve angle of the Bowden cable. According to the three friction modes, the main force transmission varies by different friction factor *μ*. Based on the experiment test, the friction factor of three friction modes (see Figure 4) is as follows: (a) *μ*_1_ = *f*_1_/*N*_1_ = 0.1, (b) *μ*_2_ = *f*_2_/*N*_2_ = 0.17, and  (c) *μ*_3_ = *f*_3_/*N*_3_ = 0.19. As for the curve angle of the Bowden cable, the length between the two sheath brackets plays a large influence on it and its friction factor.

**Figure 6:**
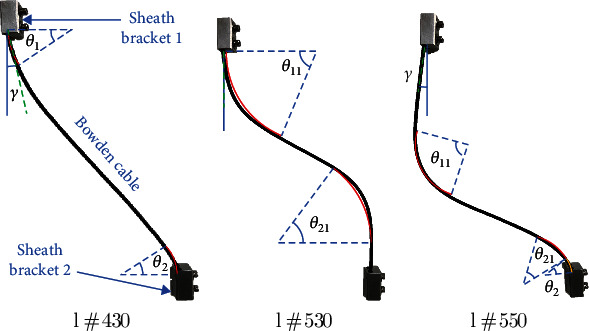
Various states with different lengths of Bowden cable in two sheath brackets.

According to the actual distances of the two sheath brackets, 13 kinds of various lengths of Bowden cable ([430 mm, 440 mm, 450 mm, 460 mm, 470 mm, 480 mm, 490 mm, 500 mm, 510 mm, 520 mm, 530 mm, 540 mm, and 550 mm]) were selected to test their curve angles on a test board.  Figure 6 illustrated 3 kinds of Bowden cable with the length of 430 mm, 530 mm, and 550 mm.

As we can see from this Figure 6(a), when the axis direction of the sheath bracket and tangential direction of the Bowden cable exists the angle *γ*, it indicates a pressure force of extrusion will produce between Bowden cable and sheath bracket, and the curve angle of this Bowden cable is *θ*_1_ and *θ*_2_. Along with the length of Bowden cable increases, the pressure force of extrusion gradually falls down; meanwhile, the values of *θ*_1_ and *θ*_2_ also decline. In contrast, the curve angle *θ*_11_ and *θ*_21_ of the Bowden cable in free state arises due to its certain stiffness of Bowden cable.  Figure 6(b) showed the Bowden cable in complete freedom, and the axis direction of the sheath bracket and tangential direction of the Bowden cable is coincided. If the length of Bowden cable continues to increase, the Bowden cable will present a sudden change of its form (see Figure 6(c)). The angle *γ* reappears, which would bring a new friction; furthermore, the angle *θ*_2_  also arises due to the extrusion between the Bowden cable and sheath bracket. It can be concluded that the friction in this phase would increase with regard to the free state.

Based on the analysis, the curve angle of *θ*_1_ + *θ*_2_ with the extrusion state and *θ*_11_ + *θ*_21_ with free state was measured according to various lengths *l* (430mm ~ 550mm) of Bowden cable (see Table 1). We neglected the value of angle below 10°, because it is too difficult to detect.

**Figure 7:**
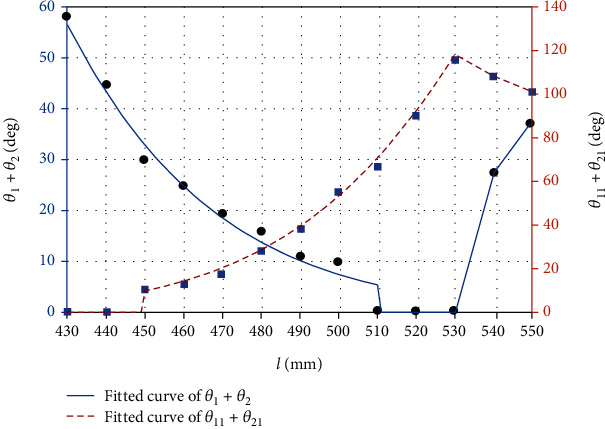
Fitting curves of different angles with various lengths of Bowden cable.

According to Table 1, when the length of Bowden cable is more than 530 mm, the angles of *θ*_1_ + *θ*_2_ and *θ*_11_ + *θ*_21_ turn into an opposite direction due to the sudden change of the Bowden cable. However, the angles of *θ*_1_ + *θ*_2_ with the length *l* from 430 mm to 510 mm and *θ*_11_ + *θ*_21_ with the length *l* from 450 mm to 530 mm can be fitted as the following equations by Gaussian approximation. The fitted curves based on the Equation (8) was illustrated in Figure 7.

(8) $$\begin{array}{c} \left\{\begin{array}{c} \theta_{1}+\theta_{2}=5902e^{-{\left(\left(l-78.69\right)/163.3\right)}^{2}},\\ \theta_{11}+\theta_{21}=563.5e^{-{\left(\left(l-661.6\right)/105.1\right)}^{2}}. \end{array}\;\right. \end{array}$$

In Equation (8) and Figure 7, the fitted curve of *θ*_1_ + *θ*_2_ reflected the variation of the length *l* from 430 mm to 510 mm because the curve angle is too small to detect with the length *l* between 510 mm and 530 mm; moreover, the fitted curve of *θ*_11_ + *θ*_21_ reflected the variation of the length *l* from 450 mm to 530 mm because the curve angle is too small to detect with the length *l* between 430 mm and 450 mm. The variation rules between the force transmission and the curve angles of Bowden cable should be found in the valid length range (450 mm~510 mm).

### 2.2. Computation and Optimization of the Force Transmission

**Figure 8:**
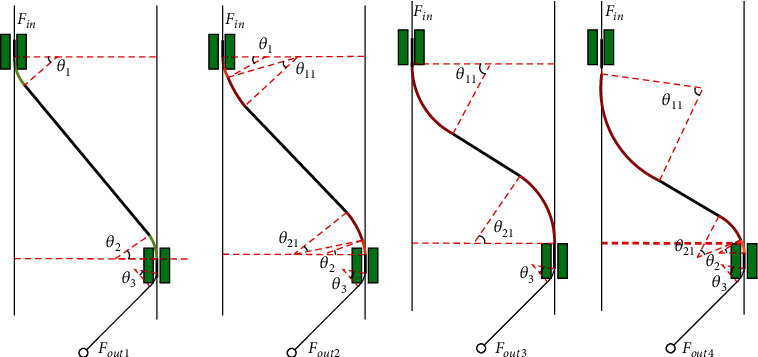
Changing process with various lengths of Bowden cable. (a) The Bowden cable mode with the angle *θ*_1_, *θ*_2_, and *θ*_3_. (b) The Bowden cable mode with the angle *θ*_1_, *θ*_2_, *θ*_11_, *θ*_21_, and *θ*_3_. (c) The Bowden cable mode with the angle *θ*_11_, *θ*_21_, and *θ*_3_. (d) The Bowden cable mode with the angle *θ*_2_, *θ*_11_, *θ*_21_, and *θ*_3_.

According to the fitted curves and Equations (8) above, we obtained the variation pattern of Bowden cable curve angles with length changes. Based on this association, the force transmission efficiency of Bowden cable can be calculated and analyzed. First, the Bowden cable between two sheaths under various lengths was shown as Figure 8. As we can see from this figure, there are four modes of Bowden cables illustrated based on the increasing length.

According to the four diagrams of Figure 8, the segment equations between *F*_out_ and *F*_in_  can be obtained based on Equation (7) and various friction factors due to the series feature of the force transmission, and we suppose the transmission velocity as a consistent direction. Then the segment force transmission models were obtained as follows:

(9) $$\begin{array}{c} \left\{\begin{array}{c} F_{\text{out}1}=F_{\text{in}}e^{-\mu_{2}\left(\theta_{1}+\theta_{2}\right)-\mu_{3}\theta_{3}},\\ F_{\text{out}2}=F_{\text{in}}e^{-\mu_{2}\left(\theta_{1}+\theta_{2}\right)-\mu_{1}\left(\theta_{11}+\theta_{21}\right)-\mu_{3}\theta_{3}},\\ F_{\text{out}3}=F_{\text{in}}e^{-\mu_{1}\left(\theta_{11}+\theta_{21}\right)-\mu_{3}\theta_{3}},\\ F_{\text{out}4}=F_{\text{in}}e^{-\mu_{2}\theta_{2}-\mu_{1}\left(\theta_{11}+\theta_{21}\right)-\mu_{3}\theta_{3}}, \end{array}\;\right. \end{array}$$

where  *F*_in_  is the force at the point of the sheath bracket 1 provided by the motor, *θ*_3_ is the curve angle of the wirerope through the sheath bracket in front of the thigh; furthermore, the value of *θ*_3_ is considered as 45° based on the maximum bending angle of knee. *F*_out1_ is output force acting on the anchor of knee when *l* = 430mm, *θ*_1_ = 30°, and *θ*_2_ = 28°, which is the minimum length condition of Bowden cable. With the length increasing of the Bowden cable, the value of *θ*_1_ + *θ*_2_ decreases while the value of *θ*_11_ + *θ*_21_ arises and adds up. The force *F*_out2_ is depended on the various curve forms and their friction factors, what is more, the force *F*_out3_  is the limit value of *F*_out2_ when the Bowden cable is just under the free state. When the length of Bowden cable continues to increase, there will be a sudden change in the trend form. At this time, the sum of bending angles of Bowden cable in the free status will decrease. The extrusion with the lower sheath bracket will cause  *θ*_2_ to reappear. The force *F*_out4_ is computed under the two conditions: (1) *l* = 540mm, *θ*_2_ = 28°, *θ*_11_ + *θ*_21_ = 108°; (2) *l* = 550mm, *θ*_2_ = 37°, *θ*_11_ + *θ*_21_ = 100°.

**Figure 9:**
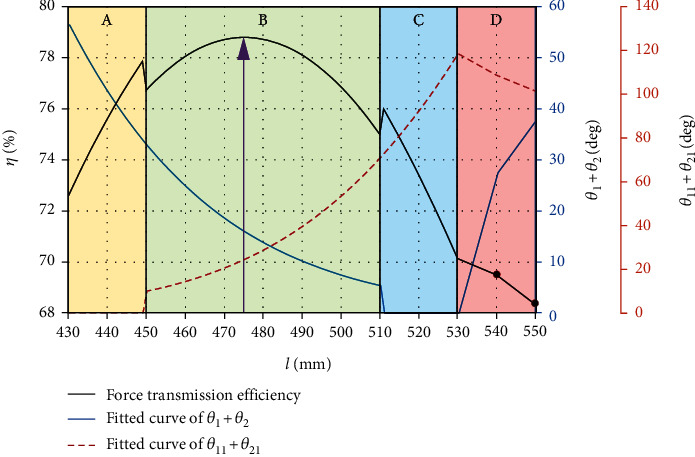
The relationship between *η* and variation of Bowden cable.

Based on the above piecewise modeling of transmission force, we obtained the corresponding change of transmission force during the change of Bowden cable length. In the transmission force model of Bowden cable, the ratio of the output force (*F*_out1_ ~ *F*_out4_) at the end of the Bowden cable to the input force (*F*_in_) at the beginning of the Bowden cable is the transmission efficiency (*η*) of the Bowden cable. The relationship between the change process of *η* and length and curve angle of Bowden cable is shown in Figure 9.

Figure 9 illustrated the relation of force transmission efficiency and variation of Bowden cable, which was divided into four regions consistent with Equation (9). There exists a sudden change from region A to region B due to neglecting the angle of *θ*_11_ + *θ*_21_ in region A and also exists another sudden change from region B to region C due to neglecting the angle of *θ*_1_ + *θ*_2_  in region C. We can conclude that the actual force transmission value may be lower than the value of region A and region C. However, the value from region B reflected the complete curve angles of *θ*_1_ + *θ*_2_ and *θ*_11_ + *θ*_21_, which indicated the efficient data. Furthermore, the efficiency data from region D only illustrated two points when the length of Bowden cable is 540 mm and 550 mm.

As we can see from Figure 9, when the length of Bowden cable is 430 mm, the extrusion between sheath bracket and Bowden cable brings transmission friction, and its transmission efficiency is about 72.59%. As the length of Bowden line increases, the extrusion force decreases, then the transmission efficiency increases gradually. When the length of Bowden cable is 450 mm, the curve angle *θ*_11_  and *θ*_21_ of Bowden cable in the free state appears, as shown in Equation (8), it can also bring some friction loss although which is smaller than the fiction loss of extrusion between sheath bracket and Bowden cable. Because *θ*_11_ + *θ*_21_  gradually increases and *θ*_1_ + *θ*_2_ gradually decreases, transmission efficiency of the Bowden cable is still increasing. When the length of Bowden cable is 475 mm, *θ*_11_ + *θ*_21_ = 24°, *θ*_1_ + *θ*_2_ = 16.3°, its transmission efficiency is 78.68%, which is the maximum value in the process. As the length of Bowden cable continues to increase, the curve angle in free state increases rapidly, and the transmission efficiency begins to decline due to the continuous increase of friction. When the length of Bowden cable is 530 mm, its transmission efficiency is down to 70.2%. When the Bowden cable length continues to increase, because of the slow attenuation of curve angle in free status and the extrusion of Bowden cable and sheath bracket appearing again, the force transmission efficiency is further reduced. According to the data fitting and simulation above, we can conclude that when the length of Bowden cable is 475 mm, the transmission efficiency reaches the maximum value under this condition.

## 3. Experiments and Results

### 3.1. Force Transmission Experiment Device

**Figure 10:**
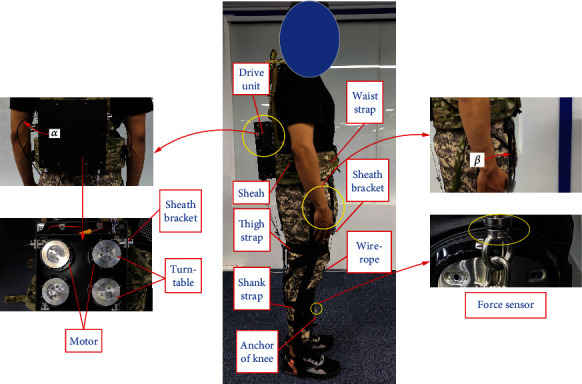
A flexible exoskeleton for the experiment test.

**Figure 11:**
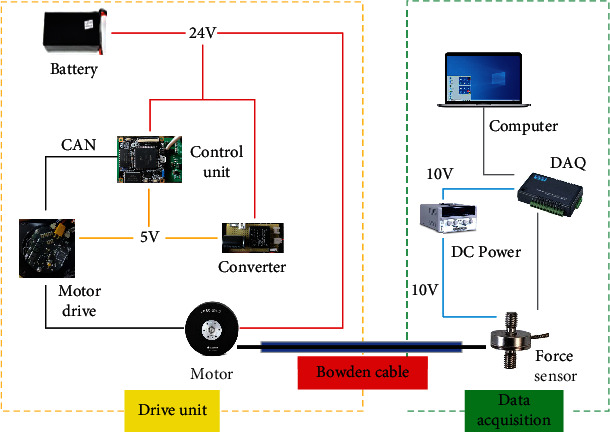
The schematic diagram of the flexible exoskeleton electrical system.

To verify the force transmission model and its efficiency in the simulation of Bowden cable, a lower limb assisting test device of the flexible exoskeleton was assembled (see Figure 10). As shown in this figure, the flexible exoskeleton was designed to assist the extension of hip and knee joints. The drive unit was installed at the back and restrained by the waist strap, which consisted of four motors, a power supply and several control modules. The model of motors was AK80-6 (12 Nm, 24 V, T-motor), which showed a disc form. One side of the wire rope was fixed on the turntable which was fastened on the motor, and the other side of the wire rope entered the sheath of Bowden cable, then was connected to the anchor of the knee. The whole sheath of Bowden cable was restrained between the two sheath brackets, which presented two curve angles *α* and *β* at the beginning and the end of the sheath. The extension of the knee can be assisted when the motor dragged the Bowden cable, and the tension transducers (LCM201, 500 N, OMEGA) were fixed on the two ends of the Bowden cable, which produced the real-time data of the input and output force values. The core control unit adopted the STM32F429IGT6 system (cortex-m4, STMicroelectronics), which transferred the instructions to the motor driver through CAN communication. The data acquisition system transmits the data to the upper computer for processing through a USB-4716 acquisition card (16bits, ADVANTECH).  Figure 11 is the schematic diagram of the flexible exoskeleton electrical system.

According to the experiment device above, the force transmission efficiency can be obtained with the variation of input force and output force by the force sensors. The testee wears the flexible exoskeleton, and his leg presented a limit swing phase, which was consistent with the state of Bowden cable modeling. This study was approved by the Ethics Committee of North China University of Technology. The participants signed an informed consent form to participate in the study.

### 3.2. Force Transmission Experiment Data Analysis

Two kinds of force efficiency experiment were developed: (1) force transmission with various lengths of Bowden cable; (2) force transmission with various motor's velocities.

#### 3.2.1. Various Lengths of Bowden Cable

**Figure 12:**
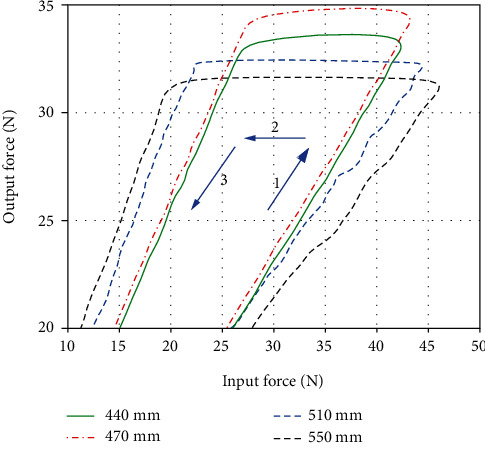
The variation of force transmission with various lengths of Bowden cable.

Four lengths of Bowden cable were selected for the efficiency experiment, which are 440 mm, 470 mm, 510 mm, and 550 mm. A spring was fixed at the end of the Bowden cable, which provided a preload with 20 N. The output velocity of the motor was set as 20 r/min. After a closed loop stroke of the spring's stretching and retracting for four Bowden cables, the input force and output force of the Bowden cable were detected by force sensors as Figure 12. As we can see from this figure, there exist three phases of the force transmission: (1) phase 1 showed the positive tension process for assisting the knee extension; (2) phase 2 illustrated a moment of the motor steering switch, and the force from the motor declined instantly; (3) phase 3 presented the reversed tension process with the oppositive velocity of Bowden cable. According to the phase 1, the output force in phase 3 is larger than the input force due to the spring initiative.

**Figure 13:**
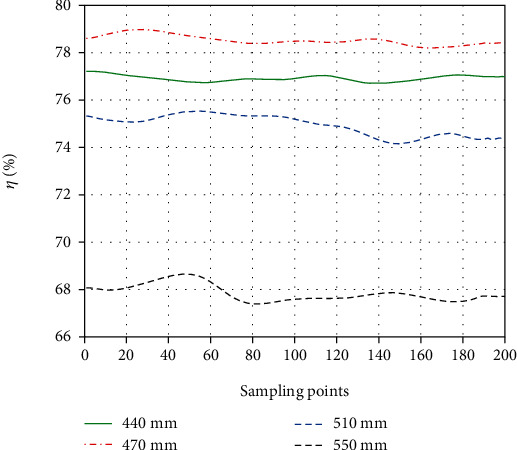
The variation of efficiencies with various lengths of Bowden cable.

As for our experiments, the assisting process (phase 1) is the most focus area. Then, 200 sampling points of input force and output force were extracted for calculating the efficiencies of the four lengths of Bowden cable (see Figure 13).

Using Figure 13, corresponding efficiencies with different lengths of Bowden cable were obtained. The efficiencies with 440 mm, 470 mm, 510 mm, and 550 mm are about 77%, 78.5%, 75.3%, and 67.8%, and the errors are 1.87%, 0.26%, 0.53%, and 1.03% compared to the simulation calculation with Figure 9, which showed approximate results.

#### 3.2.2. Various Transmission Velocities

**Figure 14:**
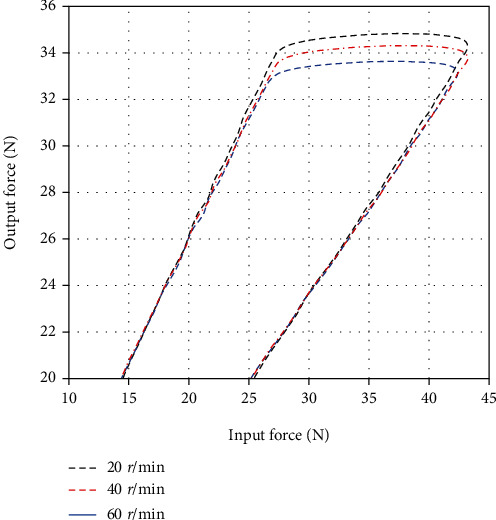
The variation of force transmission with various velocities of motor.

In the force transmission of Bowden cable, the length and its curve angle usually play major influences in its efficiency. To investigate the effect of transmission velocity of Bowden cable, we conducted a series of test experiment. The experiment of different transmission velocities can be set by the motor control; then, the output velocity of 20 r/min, 40 r/min and 60 r/min with the motor was tested to examine the velocity influence on transmission efficiency (see Figure 14). The Bowden cable with length of 470 mm was used as the same test condition.

**Figure 15:**
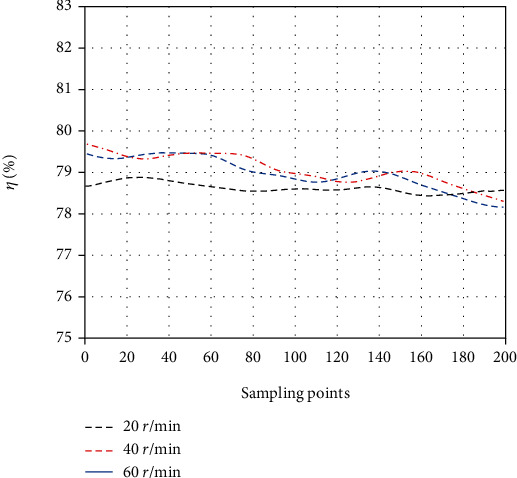
The variation of efficiencies with various velocities of motor.

As we can see from Figure 14, the input force and output force are similar in phase 1, and the efficiency *η* of the force transmission with three kinds of velocity illustrated a small oscillation in the range between 78.5% and 79.5% (see Figure 15). It can be concluded that the force transmission efficiency has less relation to the transmission velocity.

As demonstrated by the above experiment data analysis, the force transmission efficiency has large association with the length and curve angle of Bowden cable on body, instead of the transmission velocity. The optimum efficiency is about 78.5% with the length of 470 mm and the velocity of 20 r/min, which presented similar result with the modeling and simulation of the Bowden cable.

### 3.3. Metabolic Cost Experiment and Analysis

**Figure 16:**
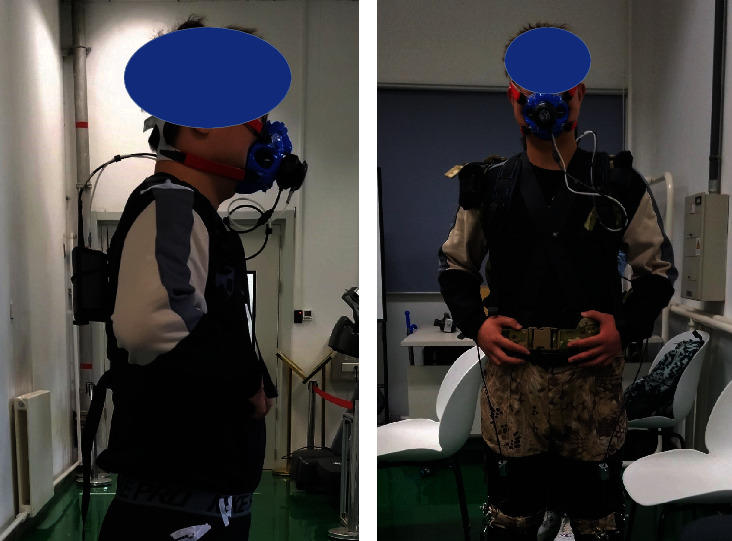
The testee wearing a respirometer for the metabolic cost test. (a) The testee without exoskeleton. (b) The testee with exoskeleton.

**Figure 17:**
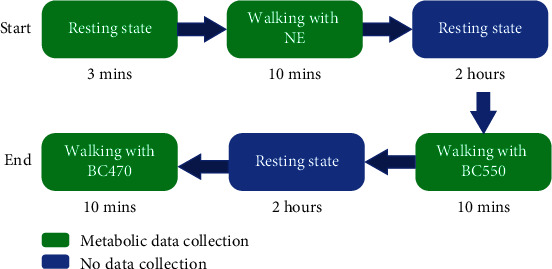
The process of metabolic cost test.

The net metabolic cost of human walking with a certain load is an effective method to evaluate the exoskeleton assistance. The exoskeleton with different lengths of Bowden cable and no exoskeleton was set as the experiment conditions. The mass of the load along with walking was set as 20 kg, and the walking speed was set as 5 km/h by using a treadmill (DT30, JOROTO). 5 testees (height: 1.75 ± 0.03 m, mass: 72 ± 2.6 kg) participated in this experiment test. A respirometer (K5, COSMED) was used to record their oxygen consumption and carbon dioxide expulsion during experiment test (see Figure 16). The process of the experiment test is showed as Figure 17.

**Figure 18:**
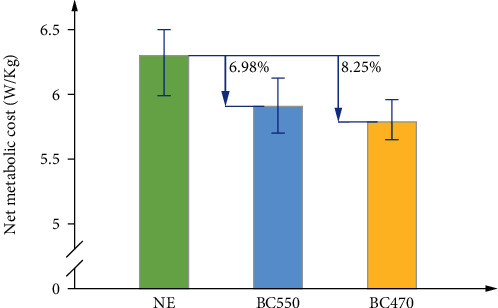
The net metabolic cost comparison of three test conditions.

As we can see from Figure 17, first of all, every testee needs to walk with no exoskeleton (NE) during 10 minutes after a short resting state; then, they have a second rest with 2 hours. Second, the testees need to walk with exoskeleton by using the Bowden cable with the length of 550 mm (BC550mm) during 10 minutes; furthermore, the Bowden cable with the length of 470 mm (BC470mm) was adopted to test after another rest with 2 hours. The steps in this process with green blocks in Figure 17 need to be collected the metabolic cost data. The net metabolic cost is calculated with the equation from the Re [37]. The statistical data of the experiment test results are illustrated as Figure 18.

As we can see from Figure 18, the average values of net metabolic cost are 6.3 ± 0.148 W/kg, 5.91 ± 0.116 W/kg, and 5.78 ± 0.108 W/kg (mean ± SEM) with NE, BC550, and BC470. Compared with NE condition, the average net metabolic cost declined by 6.98% and 8.25% with BC550 and BC 470. The two-side paired *t*-test was adapted, and the significance level was set as 0.05. According to the statistical data, there existed a significant reduction between NE and BC550 (*P* = 0.011), and a larger reduction between NE and BC470 (*P* = 0.0016). However, the reduction between BC550 and BC470 presented weak significance (*P* = 0.222). It indicated the effectiveness of our exoskeleton; furthermore, the optimal Bowden cable also provided the more efficient power and less metabolic cost.

## 4. Conclusions

Based on the results from the study of force transmission with the Bowden cable above, an association between the transmission efficiency and the lengths or curve angles of Bowden cable on body was discovered. The force transmission and metabolic cost experiments verified the analysis and optimization of Bowden cable. The main conclusions are listed as following:

1. The flexible exoskeleton is a wearable robot, which indicated that the lighter the self-weight, the better the wearable. The high transmission efficiency is a key factor for weight loss, and it is essential to analyze the Bowden cable transmission which is the main influence factor to the assisting effect
2. The transmission characteristic of Bowden cable on body showed quite different from that of the experiment test board. The friction between wire rope and sheath of Bowden cable in extrusion state, the friction between the wire rope and the sheath bracket both played significantly higher influence on the force transmission
3. The segment force transmission models of Bowden cable with various friction factors between the two sheath brackets were established, based on which, the association between the variation of the lengths of Bowden cable and the efficiency of force transmission was obtained. Furthermore, the simulation result showed that the optimum efficiency was 78.68% when the length of Bowden cable between the two sheath brackets was 475 mm
4. A flexible exoskeleton prototype was assembled to evaluate the theoretical analysis, and the experiment results suggested that the force transmission efficiency had a strong relationship to the length or curve angle of the Bowden cable, instead of the transmission velocity. The optimal efficiency and length of Bowden cable from this prototype experiment showed consistency with the modeling and simulation in this paper
5. The metabolic cost test not only presented the reduction of human consumption with the knee assistance but also indicated that the effectiveness of our exoskeleton and its optimization of the Bowden cable

## Figures and Tables

**Table 1:** The lengths of Bowden cable and corresponding curve angles.

*l* (mm)	*l* = 430	*l* = 440	*l* = 450	*l* = 460	*l* = 470	*l* = 480	*l* = 490	*l* = 500	*l* = 510	*l* = 520	*l* = 530	*l* = 540	*l* = 550
*θ*_1_ + *θ*_2_ (deg)	58	45	30	26	19	17	11	10	0	0	0	28	37
*θ*_11_ + *θ*_21_ (deg)	0	0	11	13	19	28	40	56	68	91	118	108	100

## Data Availability

The data used to support the findings of this study are available from the corresponding author upon request.
